# Cerebral Venous Thrombosis Presenting With Binocular Blindness and Bilateral Sensorineural Hearing Loss

**DOI:** 10.7759/cureus.79486

**Published:** 2025-02-22

**Authors:** Lhara Monique L Llapitan, Herminigildo H Gan

**Affiliations:** 1 Department of Neurology, Jose R. Reyes Memorial Medical Center, Manila, PHL

**Keywords:** cerebral venous sinus thrombosis (cvst), cerebrovascular disease, intracranial hypertension, oral contraceptive pills, stroke

## Abstract

Cerebral venous thrombosis (CVT) is caused by occlusion of dural sinuses and cerebral and cerebellar veins. It is a rare type of stroke usually presenting with headache, seizure, and focal motor deficit. We report a case of a 27-year-old Asian female with no significant medical history but a four-year history of unsupervised use of oral contraceptives, who presented with a sudden-onset headache followed by a generalized tonic-clonic seizure. Neurologic examination revealed papilledema with light perception in both eyes, right lateral rectus palsy, and bilateral sensorineural hearing loss. Imaging studies showed a subacute convexal subarachnoid hemorrhage over the right parietal lobe and thrombosis in the posterior superior sagittal sinus. She was given levetiracetam for seizure control and dabigatran for anticoagulation. Upon discharge, her hearing loss and lateral rectus palsy had resolved; however, bilateral blindness persisted.

This case highlights the importance of recognizing rare manifestations of cerebral venous thrombosis, such as bilateral blindness and sensorineural hearing loss. These symptoms can be overlooked due to the more common presentations of headache, seizure, and focal motor deficits.

## Introduction

Cerebral venous thrombosis (CVT) is a type of cerebrovascular disease caused by occlusion of cerebral and cerebellar veins, or dural sinuses. About 0.5-1.0% of all strokes are caused by CVT [1]. It may occur at any age with a peak incidence at 20-50 years and a 3:1 female preponderance [1]. Patients with CVT might present with a wide range of symptoms that are acute and progressive. Approximately 60-90% of patients reported having severe headaches, which is the most prevalent and typically the initial sign of CVT [2]. Around 30-40% of patients have acute symptomatic seizures, while 30-50% present with focal motor deficits [2]. We report a case of cerebral venous thrombosis presenting with both binocular blindness and bilateral sensorineural hearing loss.

## Case presentation

A 27-year-old Asian female who was previously well with no known comorbidities presented at our emergency department in September 2024 after a sudden onset of moderate-to-severe headache, followed by a generalized tonic-clonic seizure. There were no other symptoms such as vomiting and blurring of vision. She has no smoking history but has been taking oral contraceptives (ethinyl estradiol+levonorgestrel 30 mcg/125 mcg) for the past four years, unsupervised by her gynecologist. A review of her family history was non-contributory.

Upon examination, the patient was drowsy, easily arousable by light tapping, oriented to three spheres, and was able to follow commands. On ophthalmologic examination, the patient was only able to perceive light in both eyes, with pupils 2-3 mm equally and briskly reactive to light and with papilledema on fundoscopic examination. On neurologic examination, she had lateral rectus palsy on the right and bilateral sensorineural hearing loss. Upon general physical examination, vital signs were within normal limits and the patient was noted to be obese with a BMI of 30 [3]. The rest of her neurologic and general physical examination findings were unremarkable.

**Figure 1:**
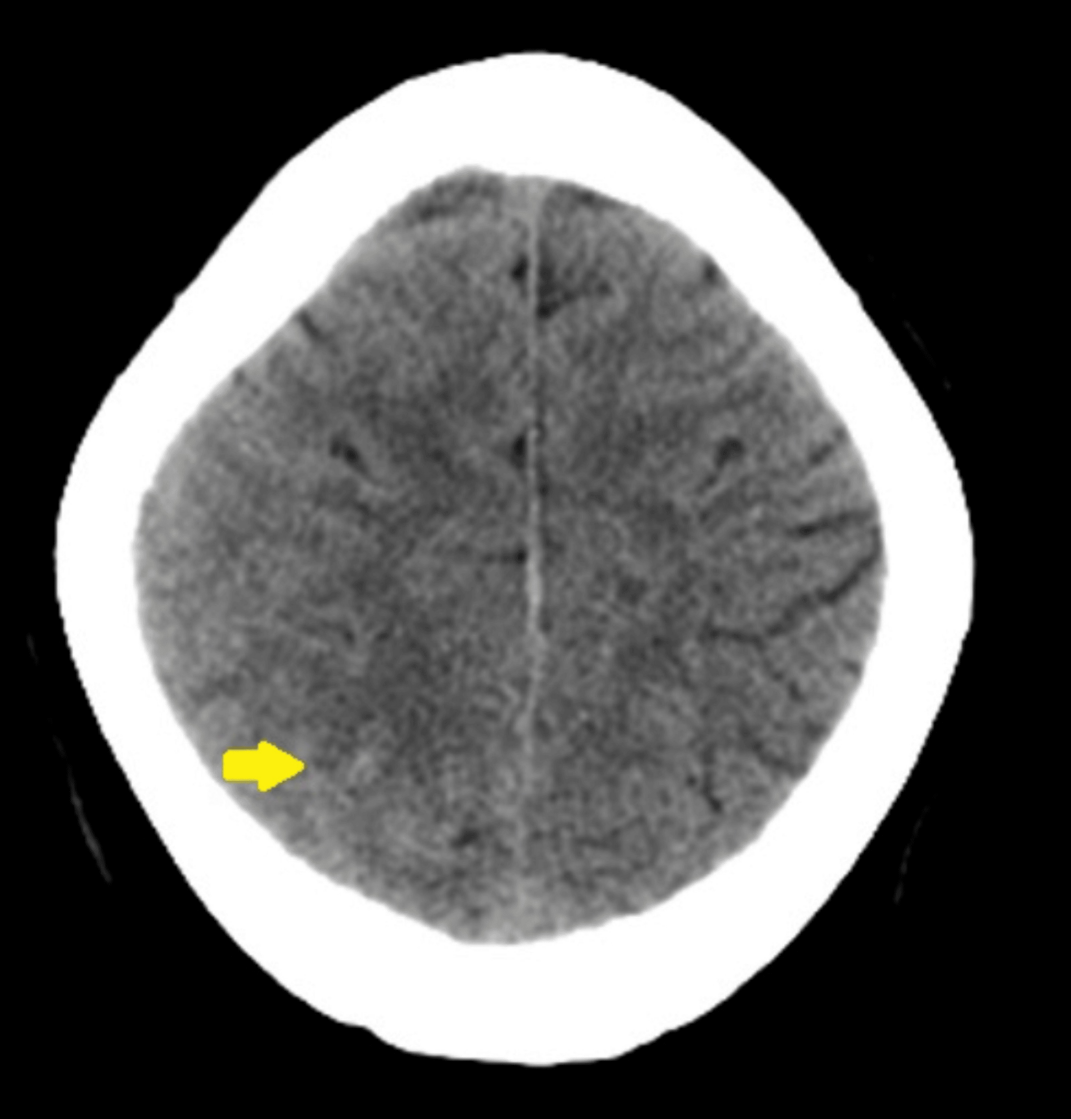
Axial non-contrast enhanced cranial CT scan taken one-day post-ictus. The CT scan revealed acute convexal subarachnoid hemorrhage over the right parietal lobe (arrow).

**Figure 2:**
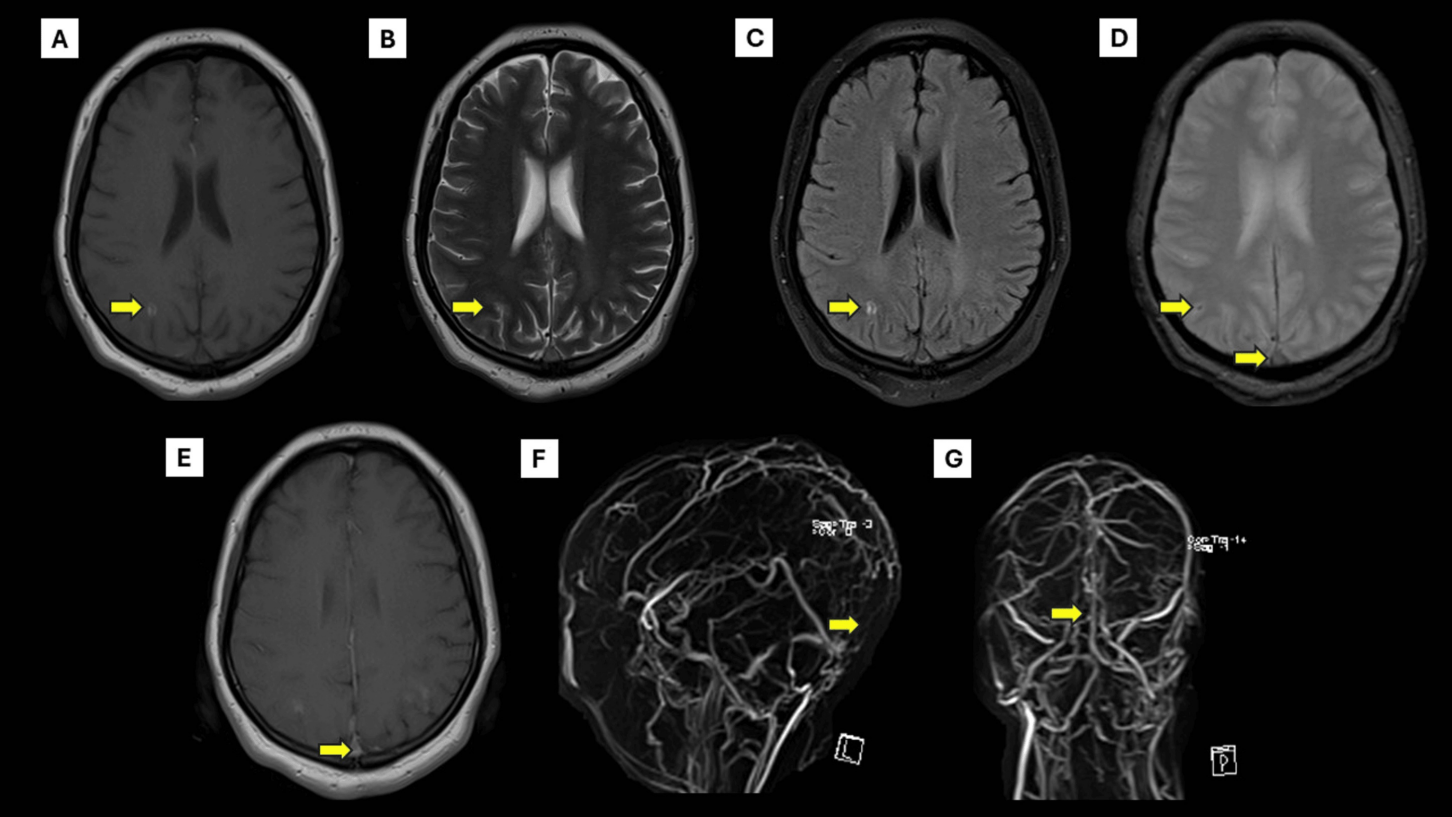
Cranial MRI with MRV findings. Axial T1-weighted (A), T2-weighted (B), T2 fluid-attenuation inversion recovery (FLAIR) (C), and gradient-recalled echo (GRE) (D) magnetic resonance imaging (MRI) taken one-day post-ictus, revealed a subacute convexal subarachnoid hemorrhage (arrow). Axial GRE sequence (D) and T1-weighted, post-gadolinium MR image (E) showed an empty delta sign, signifying superior sagittal sinus thrombosis (arrow). MR venography (F, G) revealed a filling defect in the posterior portion of the superior sagittal sinus, which may also signify thrombosis (arrow). MRV: magnetic resonance venography

**Figure 3:**
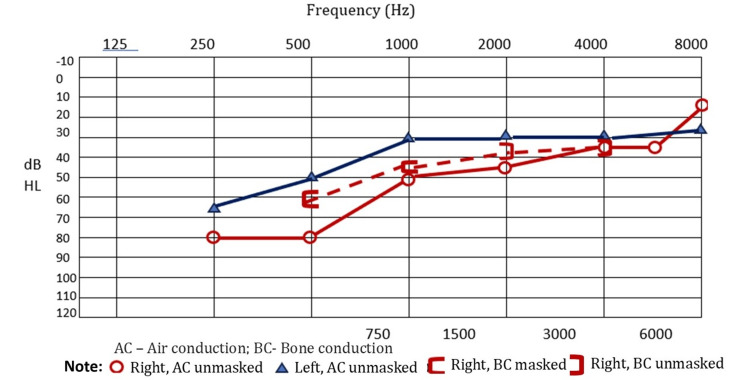
Pure tone audiometry. Moderate sensorineural hearing loss rising to mild at higher frequencies on the left and moderate-to-severe mixed hearing loss rising to moderate at higher frequencies on the right.

Initial blood counts, coagulation parameters (prothrombin time, partial thromboplastin time, bleeding and clotting time), biochemical laboratory tests, and the initial ECG were all normal. D-dimer was markedly elevated at 1,300 mg/L (normal: <0.5 mg/L). The cranial computed tomography (CT) scan and subsequent cranial magnetic resonance (MR) imaging revealed subarachnoid hemorrhage over the right parietal convexity (Figures 1, 2A-2D). Additionally, contrast-enhanced MR imaging (Figure 2E) and MR venography revealed thrombosis of the posterior portion of the superior sagittal sinus (Figures 2F, 2G). A fundus photo revealed obscuration of all major vessels on the disc, leaving the disc with complete elevation of the disc, and cup showing a circumferential halo. Optical coherence tomography (OCT) showed increased disc size in both eyes. Pure tone audiometry (PTA) revealed moderate sensorineural hearing loss rising to mild at higher frequencies on the left and moderate-to-severe mixed hearing loss rising to moderate at higher frequencies on the right (Figure 3). Findings are consistent with cerebral venous thrombosis of the superior sagittal sinus.

Oral contraceptives were discontinued. Antiseizure medication (levetiracetam 500 mg/tab twice a day) and anticoagulation (dabigatran 150 mg twice a day) were initiated. Lumbar drainage was also placed with an elevated opening pressure at 21 cmH_2_0, which had already normalized in the subsequent days; hence, it was removed after five days. She was discharged after a two-week admission with improved sensorium. She was awake, conversant, and oriented to three spheres, and can follow commands. There is also resolution of hearing loss and lateral rectus palsy, though bilateral blindness persisted. Repeat fundoscopic examination revealed the atrophy of both optic nerves. She was discharged with dabigatran 150 mg twice daily for six months, and levetiracetam 500 mg twice daily. On the three-month follow-up, the neurologic examination was unchanged, with no seizure recurrence.

## Discussion

Cerebral venous thrombosis is caused by occlusion of dural sinuses and cerebral and cerebellar veins. Its estimated incidence ranges from 0.2 to 1.32 per 100,000 person-years [1]. Typical symptoms include headaches, seizures, altered mental status (from mild disorientation to coma), and motor paralysis in the limbs in 40% of patients [4,5].

CVT is grouped into the following four clinical syndromes: isolated intracranial hypertension usually presenting as headache, papilledema, tinnitus and decreased visual acuity, focal syndrome with the presence of neurologic deficit, often with seizure, diffuse encephalopathy which may be secondary to thrombosis of deep venous system, and cavernous sinus syndrome which lead to orbital pain, proptosis, chemosis as well as ophthalmoplegia [2].

Papilledema, or swelling of the optic disc as a result of elevated intracranial pressure, is one of the most common visual problems in CVT [5]. The overall incidence of permanent blindness in CVT is reported to be low, ranging between 3% and 13% among survivors. It may be secondary to a raised intracranial pressure which may lead to optic nerve dysfunction. Other mechanisms may include bilateral occipital infarcts due to compression of posterior cerebral artery in those with uncal herniation secondary to a large venous infarction. To reduce the likelihood of irreversible visual outcomes, patients with CVT must have regular visual function monitoring [5,6].

CVT rarely presents as sensorineural hearing loss. Moreover, those having sensorineural hearing loss are mostly unilateral, and bilateral involvement is only less than 5% [5]. In a retrospective analysis examining the prevalence of CVT individuals presenting with sensorineural hearing loss (SSNHL), CVT was detected in only two of the 554 SSNHL patients. This implies that although SSNHL is not commonly seen, it may be a presenting symptom of CVT. The mechanism behind this symptom may be explained by a number of processes, including high intracranial pressure secondary to a venous blockage, which alters inner ear fluid drainage, and perhaps impairs hearing. Cochlear injury may be directly caused by compression of the vestibulocochlear nerve or indirectly caused by the transmission of increased intracranial pressure to the endolymph [7,8]. Following anticoagulant treatment, full hearing recovery can be achieved. In general, hearing loss is rare in CVT. When it does happen, it is frequently unilateral and, with the right care, reversible [9,10,11].

We discussed a case of cerebral venous thrombosis presenting with both binocular blindness and bilateral sensorineural hearing loss. Neuroimaging such as CT or MRI is usually the initial test requested, especially when faced with non-specific acute presentations. It may raise suspicion upon seeing the following findings: visualization of the thrombus, venous infarction with or without hemorrhagic transformation, and absence of venous filling. On non-contrast CT, various radiologic signs may suggest the presence of a thrombus, including the dense vessel sign, cord or string sign, and empty delta sign. These signs can be observed up to 14 days after the onset of symptoms. On cranial MRI, the signal intensity, particularly of T1 and T2, may change depending on the age of hematoma. Gradient-recalled echo would then be useful since thrombosis may present as a blooming artifact. To confirm the diagnosis of CVT, CT, or MR venography may be utilized wherein thrombi may present as filling defects [1].

The primary treatment for cerebral venous thrombosis is anticoagulation, typically with heparin or low-molecular-weight heparin with initial doses given either intravenously (IV) or subcutaneously (SC) for three to five days, then transition to oral vitamin K antagonists (VKAs) for three to six months [12,2]. A multicenter international retrospective study shows that direct oral anticoagulant (DOAC) particularly dabigatran 150 mg/tab BID, taken for six months, is another safe and effective alternative option to VKAs [13]. In another multicenter, retrospective observational study conducted to assess the efficacy and safety of apixaban in treating CVT, apixaban (dosed between 2.5 mg and 5 mg twice daily) was compared to vitamin K antagonists (VKAs), with no significant differences found in the outcomes. However, this study excluded patients with active malignancy and antiphospholipid syndrome. Moreover, those with factor V deficiency were treated with VKA, not apixaban [14]. In the randomized controlled feasibility study (SECRET trial), the use of rivaroxaban, another oral anticoagulant, was investigated as a treatment for CVT. The study used a simplified regimen of rivaroxaban 20 mg once daily, and compared it in terms of its feasibility, safety, and efficacy, against the standard-of-care anticoagulation which consisted of either warfarin or low-molecular-weight heparin. For its safety profile, no deaths were reported; however, one primary outcome event occurred in the rivaroxaban group, namely, a symptomatic intracranial hemorrhage. Also, two clinically relevant non-major bleeding events also occurred, one in each treatment arm. The downside of this trial is that the recruitment rate was 21.3 participants per year, with 57% of eligible candidates consenting to participate. Hence, due to the small sample size, there are no definitive conclusions about the relative safety of rivaroxaban compared to the standard of care. The same study found improvements in patient-reported outcomes over time (modified Rankin Scale, quality of life, headache, mood, fatigue, and cognition), with no significant difference between the rivaroxaban and the control group. However, the small sample size limits the power of this finding. Hence, a larger trial is needed to confirm these findings [15]. An endovascular intervention such as mechanical thrombectomy in the context of CVT is recommended as a last resort in cases of rapid deterioration despite anticoagulation. And in cases of large hemispheric venous infarcts, surgical decompression is lifesaving [4]. Intracranial hypertension in CVT can be treated using the following methods: osmotic therapy, hyperventilation, and head elevation. Other interventions include lumbar puncture, especially in cases of isolated intracranial hypertension without significant cerebral edema or mass effect; carbonic anhydrase inhibitors, such as acetazolamide; and corticosteroids in cases of concomitant inflammatory conditions [16].

## Conclusions

We present the first reported case of CVT presenting with bilateral blindness and sensorineural hearing loss, consistent with a clinical syndrome of intracranial hypertension. Prompt diagnosis and treatment are necessary to prevent irreversible complications, such as in our case, permanent blindness, and complete deafness.
